# Interleukin-18 as an *in vivo *mediator of monocyte recruitment in rodent models of rheumatoid arthritis

**DOI:** 10.1186/ar3055

**Published:** 2010-06-16

**Authors:** Jeffrey H Ruth, Christy C Park, M Asif Amin, Charles Lesch, Hubert Marotte, Shiva Shahrara, Alisa E Koch

**Affiliations:** 1 Department of Internal Medicine, University of Michigan Medical School, 109 Zina Pitcher Drive, Ann Arbor, MI 48109, USA; 2 Department of Internal Medicine, Northwestern University Feinberg School of Medicine, 240 E. Huron, Chicago, IL 60611, USA; 3 Department of Internal Medicine, Ann Arbor Veteran's Administration, 2215 Fuller Road, Ann Arbor, MI 48109, USA

## Abstract

**Introduction:**

The function of interleukin-18 (IL-18) was investigated in pertinent animal models of rodent rheumatoid arthritis (RA) to determine its proinflammatory and monocyte recruitment properties.

**Methods:**

We used a modified Boyden chemotaxis system to examine monocyte recruitment to recombinant human (rhu) IL-18 *in vitro*. Monocyte recruitment to rhuIL-18 was then tested *in vivo *by using an RA synovial tissue (ST) severe combined immunodeficient (SCID) mouse chimera. We defined monocyte-specific signal-transduction pathways induced by rhuIL-18 with Western blotting analysis and linked this to *in vitro *monocyte chemotactic activity. Finally, the ability of IL-18 to induce a cytokine cascade during acute joint inflammatory responses was examined by inducing wild-type (Wt) and *IL-18 *gene-knockout mice with zymosan-induced arthritis (ZIA).

**Results:**

We found that intragraft injected rhuIL-18 was a robust monocyte recruitment factor to both human ST and regional (inguinal) murine lymph node (LN) tissue. *IL-18 *gene-knockout mice also showed pronounced reductions in joint inflammation during ZIA compared with Wt mice. Many proinflammatory cytokines were reduced in *IL-18 *gene-knockout mouse joint homogenates during ZIA, including macrophage inflammatory protein-3α (MIP-3α/CCL20), vascular endothelial cell growth factor (VEGF), and IL-17. Signal-transduction experiments revealed that *IL-18 *signals through p38 and ERK½ in monocytes, and that *IL-18*-mediated *in vitro *monocyte chemotaxis can be significantly inhibited by disruption of this pathway.

**Conclusions:**

Our data suggest that *IL-18 *may be produced in acute inflammatory responses and support the notion that *IL-18 *may serve a hierarchic position for initiating joint inflammatory responses.

## Introduction

Interleukin-18 (IL-18) is a type-1 cytokine associated with proinflammatory properties. IL-18 is present at increased levels in serum and in the rheumatoid synovium, as well as in the bone marrow in many human rheumatologic conditions, including rheumatoid arthritis (RA), juvenile RA, adult-onset Still disease, and psoriatic arthritis [1-27]. Interestingly, rheumatoid nodules have features of type-1 (Th_1_) granulomas [1,28,29] with abundant expression of type-1 inflammatory cytokines, including interferon-γ (IFN-γ) and IL-18 [1,30]. IL-18 also induces the release of type 1 cytokines by T cells and macrophages and stimulates production of inflammatory mediators, such as chemokines, by synovial fibroblasts or nitric oxide by macrophages and chondrocytes [31-35]. Among other cytokines, IL-18 is thought to play a pivotal role in the inflammatory cascade in patients with adult-onset Still disease by orchestrating the Th_1 _response and inducing other cytokines, such as IL-1β, IL-8, tumor necrosis factor-α TNF-α and IFN-γ [36].

We previously showed that IL-18 acts on endothelial cells to induce angiogenesis and cell adhesion [37,38]. A primary source of IL-18 is the macrophage; however, various other sources of IL-18 have been identified, including Kupffer cells, dendritic cells, keratinocytes, articular chondrocytes, osteoblasts, and synovial fibroblasts [5,37,39-45]. The IL-18 receptor (IL-18R) is similarly expressed on many cell types, including T lymphocytes, natural killer cells, macrophages, neutrophils, and chondrocytes [31,32,40,46], underscoring the pleiotropic nature of this receptor-ligand pair.

IL-18 has structural homology with IL-1, shares some common signaling pathways [37,47], and also requires the cleavage at its aspartic acid residue by IL-1-converting enzyme to become an active, mature protein [37,48,49]. Thus, IL-1 and IL-18 share many biologically similar inflammatory functions. Previous work implicated IL-18 in RA, as higher levels are present in RA compared with osteoarthritic synovial fluid (SF) and sera [5,37]. Also, IL-18 enhances erosive, inflammatory arthritis in murine models of systemic arthritis [5,37]. The influential role of IL-18 in articular inflammation was confirmed in mice lacking the *IL-18 *gene that had reduced the incidence and severity of collagen-induced arthritis (CIA), which was reversed by treatment with recombinant human (rhu) IL-18 [37,50]. With mice deficient in IL-18, CIA was less severe compared that in wild-type (Wt) mice [1,50], confirmed by histologic evidence of decreased joint inflammation and destruction. Furthermore, levels of bovine collagen-induced IFN-γ, TNF-α, IL-6, and IL-12 from spleen cell cultures were correspondingly decreased in IL-18-deficient animals [1].

Blocking of IL-18 was also tested in CIA [1,51-53]. Wt DBA-1 mice were treated with either neutralizing antibodies to IL-18 or the IL-18-binding protein (IL-18BP) after clinical onset of disease, resulting in significantly reduced joint inflammation and reduced cartilage erosion [1,53]. In streptococcal cell wall (SCW)-induced arthritis [1,54], neutralizing rabbit anti-murine IL-18 antibody suppressed joint swelling. This effect was noted early, after blockade of endogenous IL-18, and resulted in reduced joint TNF-α and IL-1 levels [1]. These studies clearly established a pathologic role for endogenous IL-18 in rodent arthritis. The effect of IL-18 was apparently independent of IFN-γ, because anti-IL-18 antibodies could equally inhibit SCW arthritis in mice deficient in IFN-γ [1,55].

This study was carried out to define better the cellular mechanisms induced by IL-18 contributing to the observed pathology in many of these rodent models. We clarified the cytokines induced by IL-18 in zymosan-induced arthritis (ZIA) by comparing cytokine levels from ZIA arthritic joints homogenized from *IL-18 *gene-knockout and Wt mice. We also defined the role of IL-18 to recruit monocytes to human RA ST and murine lymph nodes (LNs) in a severe combined immunodeficient (SCID) mouse chimera. This confirmed many of our *in vitro *chemotaxis findings showing that IL-18 induces monocyte chemotaxis, and that this migratory property is mediated by intracellular monocyte p38 and ERK½.

## Materials and methods

### Patient samples

Peripheral blood (PB) was obtained from healthy normal (NL) volunteers. STs were obtained from RA patients undergoing total joint replacement who met the American College of Rheumatology criteria for the classification of RA. All tissues were obtained with informed consent with Institutional Review Board approval.

### Monocyte isolation

PB was collected in heparinized tubes from NL adult donors. After centrifugation, the buffy coat was collected, and mononuclear cells were purified under sterile conditions on an Accu-Prep gradient at 400 *g *for 30 minutes at room temperature. Mononuclear cells collected at the interface were washed twice with PBS and resuspended in Hank's Balanced Saline Solution (HBSS) with calcium and magnesium (Life Technologies, Bethesda, MD, USA) at 2.5 × 10^6 ^cells/ml. Mononuclear cell viability was routinely greater than 98% (purity > 99%), as determined with trypan blue exclusion. Monocyte separation was done by adding 4 ml of mononuclear cells mixed with 8 ml of isolation buffer (1.65 ml 10 × HBSS in 10 ml of Percoll, pH 7.0) in a 15-ml siliconized tube. After centrifugation (400 *g *for 25 minutes at room temperature), monocytes were collected from the top layer of solution (5 mm). Monocytes were > 95% pure, and viability was >98% by trypan blue exclusion.

### *In vitro *monocyte migration assay

Chemotaxis assays were performed by using a 48-well modified Boyden chamber system, as done previously [34,35]. Stimulant (25 μl) of IL-18 was added to the bottom wells of the chambers, whereas 40 μl of human monocytes from NL PB at 2.5 × 10^6 ^cells/ml was placed in the wells at the top of the chamber. Sample groups were assayed in quadruplicate, with results expressed as cells migrated per high-power field (hpf; 400 ×). Hank's Balanced Saline Solution (HBSS) and fMLP (10^-7 ^*M*) were used as negative and positive stimuli, respectively. The rhuIL-18 used in all studies was purchased from MBL International Corp., through R & D Systems (Minneapolis, MN, USA). The endotoxin levels were < 0.1 ng/μg of rhuIL-18 protein that, in our hands, did not previously interfere with *in vitro *cell-migration experiments [37].

### Monocyte culture and lysis

PB was collected in heparinized tubes, and monocytes were isolated as described earlier and as we have done previously [56]. Monocytes were plated in six-well plates (5 × 10^6 ^cells/well) in serum-free RPMI. Cells were allowed to attach for 1 hour at 37°C. Fresh RPMI was used to rinse unattached cells. RPMI containing rhIL-18 was added to each well in a time-course manner, at time points 1, 5, 15, 30, and 45 minutes, with the last well receiving no IL-18 (0 minutes). Medium was removed, and 150 μl of cell-lysis buffer was added to each well. Plates were kept on ice for 15 minutes with occasional rocking. A cell scraper was used remove all cells, and lysates were removed to an Eppendorf centrifuge tube. Lysates were sonicated for 30 seconds, vortexed briefly, and spun at 10,000 RPM for 10 minutes. Supernatants were removed, measured for protein level (BCA protein assay; Pierce Biotechnology, Rockford, IL, USA), and the volume measured. Samples were frozen at -80°C until assayed.

### SDS-PAGE and Western blotting

Protein lysate (15 to 20 μg) from monocytes was run on SDS-PAGE and transblotted to nitrocellulose membranes by using a semi-dry transblotting apparatus (Bio-Rad, Hercules, CA, USA). Nitrocellulose membranes were blocked with 5% nonfat milk in Tris-buffered saline Tween-20 (TBST) for 60 minutes at room temperature. Blots were incubated with optimally diluted specific primary antibody in TBST containing 5% nonfat milk overnight at 4°C. Phosphorylation state-specific antibodies for ERK½ and p38 (Cell Signaling Technology Inc., Danvers, MA, USA) were used as primary antibodies. Primary antibodies used for phospho-p38 (p-p38) MAPK were rabbit anti-human Ab (9211; Cell Signaling) or p38 MAPK (for total p38) rabbit anti-human Ab (9212; Cell Signaling). For ERK½ signaling, the primary antibodies used were phospho-p44/42 MAPK (ERK½) rabbit anti-human Ab (4370; Cell Signaling) or p44/42 MAPK (for total ERK½) rabbit anti-human Ab (9102; Cell Signaling). The secondary antibody used for detection of all signaling molecules was anti-rabbit IgG, horseradish peroxidase (HRP)-linked Ab (7074; Cell Signaling). Blots were washed 3 times and incubated with the HRP-conjugated antibody (1:1,000 dilutions) for 1 hour at room temperature. Protein bands were detected by using ECL (Amersham Biosciences, Pittsburgh, PA, USA) per the manufacturer's instructions. Blots were scanned and analyzed for band intensities by using UN-SCAN-IT version 5.1 software (Silk Scientific, Orem, UT, USA).

### Transient transfection of human monocytes

Isolated human PB monocytes were plated in six-well plates at 2.5 × 10^6 ^cells/ml with serum-free RPMI 1640 medium overnight and subsequently transfected by using Lipofectin reagent (Invitrogen Inc., Carlsbad, CA, USA). ODN DNA (10 μ*M*) and Lipofectin (5 μl) were incubated separately in 100 μl of serum-free medium for 30 minutes. Solutions were mixed gently, and 880 μl of medium was added. A DNA/Lipofectin mixture was added to the preincubated monocytes with an additional incubation of ≥ 5 hours before use in chemotaxis studies. Transfection efficiencies for all ODNs used in this study were determined by counting FITC-transfected cells by fluorescence microscopy and comparing them with a DAPI label in the same cells. Transfection of ODNs peaked at 5 hours with an efficiency routinely > 80% (data not shown). For transient transfection of human monocytes, the sense and antisense ODNs that were used with subsequent rhuIL-18 stimulation for *in vivo *migration assays were ERK½ sense: ATGGCGGCGGCGGC; ERK½ antisense: GCCGCCGCCGCCAT [57]; JNK sense: GCTAAGCGGTCAAGGTTGAG; JNK antisense: GCTCAGTGGACATGGATGAG [58]; Jak2 sense: ATGGGAATGGCCTGCCTT; Jak2 antisense: AAGGCAGGCCATTCCCAT [59]; p38 sense: AGCTGATCTGGCCTACAGTT; p38 antisense: AGGTGCTCAGGACTCCATTT [60]. Transfected cells were used in *in vitro *monocyte chemotaxis studies.

### Human ST collection

STs were obtained from RA patients undergoing total joint replacement who met the American College of Rheumatology criteria for RA. Under sterile conditions, RA ST was isolated from surrounding tissue, cut into 0.5-cm^3 ^segments, and screened for pathogens before implantation. All tissues were stored frozen at -80°C in a freezing medium (80% heat-inactivated fetal bovine serum with 20% dimethyl sulfoxide, vol/vol), thawed and washed three times with PBS before insertion into mice. All specimens were obtained with IRB approval.

### Mice

Animal care at the Unit for Laboratory Animal Medicine at the University of Michigan is supervised by a veterinarian and operates in accordance with federal regulations. Wt and *IL-18 *gene knockout mice were bred in house according to the guidelines of the University Committee on the Use and Care of Animals. SCID/NCr mice were purchased from the National Cancer Institute (NCI). All mice were given food and water *ad libitum *throughout the entire study and were housed in sterile rodent microisolator caging with filtered cage tops in a specific pathogen-free environment to prevent infection. All efforts were made to reduce stress or discomfort in the animals used in these studies.

### Monocyte isolation and fluorescent dye incorporation

Human monocytes were isolated from the PB (~100 ml) of NL healthy adult volunteers and applied to Ficoll gradients, as previously described [56]. Monocyte viability and purity of cells was routinely > 90%. For *in vivo *studies, monocytes were fluorescently dye-tagged with PKH26 by using a dye kit per manufacturer's instructions (Sigma-Aldrich, St. Louis, MO, USA). Successful labeling of monocytes was confirmed by performing cytospin analysis and observing fluorescing monocytes under a microscope equipped with a 550-nm filter.

### Generating human RA ST SCID mouse chimeras

SCID mouse human RA ST chimeras represent a unique way to study human tissue *in vivo*. We used this model to study whether intragraft-administered rhuIL-18 can recruit monocytes *in vivo*. Six- to eight-week-old immunodeficient mice were anesthetized with isoflurane under a fume hood, after which a 1.5-cm incision was made with a sterile scalpel on the midline of the back. Forceps were used bluntly to dissect a path for insertion of the ST graft. ST grafts were implanted on the graft-bed site and sutured by using surgical nylon. Grafts were allowed to "take," and the sutures were removed after 7 to 14 days. Within 4 to 6 weeks of graft transplantation, rhuIL-18 was injected into grafts. Grafts injected intragraft with PBS served as a negative control. Immediately thereafter, mice were administered 5 × 10^6 ^fluorescently dye-tagged (PKH26) human PB monocytes through the tail vein. Mice were killed, and grafts were harvested 48 hours later. For all *in vivo *studies, integrated human monocytes to the implanted ST were examined from cryosectioned slides by using a fluorescence microscope and scored [61]. Murine LNs were fluorescently stained for human CD4-, CD11b/Mac-1-, CD14-, and CD19-expressing cells. For monocyte detection, the primary antibody was a mouse anti-human mAb (mouse anti-human CD11b/Mac-1 from BD Biosciences Pharmingen, San Jose, CA, USA; catalog no. 555385), followed by blocking with goat serum and the addition of a goat anti-mouse FITC-tagged secondary antibody (goat anti-mouse FITC IgG, Sigma-Aldrich; catalog no. 025K6046). Murine LN tissues were similarly stained for human lymphocyte CD4 (T-cell; primary mAb from BD Biosciences Pharmingen; catalog no. 3015A) and CD19 (B-cell; primary mAb from BD Biosciences Pharmingen; catalog no. 555410) followed with the corresponding FITC-tagged secondary antibody (Sigma-Aldrich). All sections were analyzed appropriately, and evaluators were blinded to the experimental setup.

### ZIA induction

Wt (13 mice) and *IL-18 *gene-knockout mice (12 mice) were divided into two separate groups, with one group receiving PBS and the other receiving zymosan (Sigma-Aldrich). Before the procedure, all mice were anesthetized with 0.08 ml of ketamine and subsequently received 20 μl/knee joint (both knees/mouse) of either PBS or zymosan (30 mg/ml). Mice were allowed to recover and were measured for joint circumference, as described previously [62]. Circumference measurements were taken at 24 hours for all mice, and at 48 hours for the remaining mice. After killing, all mice were bled for serum, and then the knees were taken for homogenate preparation and cytokine analysis.

### Clinical assessment of murine ZIA

Clinical parameters of ZIA mice were assessed at 24 and 48 hours after zymosan injection and included ankle circumference, as previously described for rat AIA [62]. For ankle-circumference determination, two perpendicular diameters of the joint were measured with a caliper (Lange Caliper; Cambridge Scientific Industries, Cambridge, MA, USA). Ankle circumference was determined by using the geometric formula: circumference = 2 π (√(*a*^2 ^+ *b*^2^/2)), where *a *is the laterolateral diameter, and *b *is the anteroposterior diameter.

### ZIA joint homogenate preparation

Wt and *IL-18 *gene-knockout mice were killed, and joints and serum were collected at 24 and 48 hours after zymosan administration. Only hind joints were used in the study. Joints were removed directly below the hairline and snap frozen in liquid nitrogen. All joints were stored at -80°C before processing. Each joint was thawed on ice and quickly homogenized on ice in 1 to 2 ml phosphate-buffered saline (PBS) containing a tablet of proteinase inhibitors (10-ml PBS/tablet; Boehringer Mannheim, Indianapolis, IN, USA). Homogenized tissues were centrifuged at 2,000 *g *at 4°C for 10 minutes, filtered, aliquoted, and stored at -80°C until analysis with ELISA.

### ELISA technique

ELISA assays were performed as described previously [34]. In brief, cytokine levels from ZIA mouse-joint homogenates were measured by coating 96-well polystyrene plates with anti-murine chemokine antibodies (R & D Systems, Minneapolis, MN, USA) followed by a blocking step. Cytokines measured were IL-1β IL-6, IL-17, TNF-α MCP-1/CCL2, MIP-1α/CCL3, MIP-3α/CCL20, RANTES/CCL5, and VEGF. All samples were added in triplicate, with rhuIL-18 as standard. Subsequently, biotinylated anti-human antibody and streptavidin peroxidase were added, and sample concentrations were measured at 450 nm after developing the reaction with TMB substrate.

### Statistical analysis

Statistical significance values for all studies were calculated by using the Student *t *test. Values of *P *< 0.05 were considered statistically significant.

## Results

### IL-18 is chemotactic for monocytes

**Figure 1:**
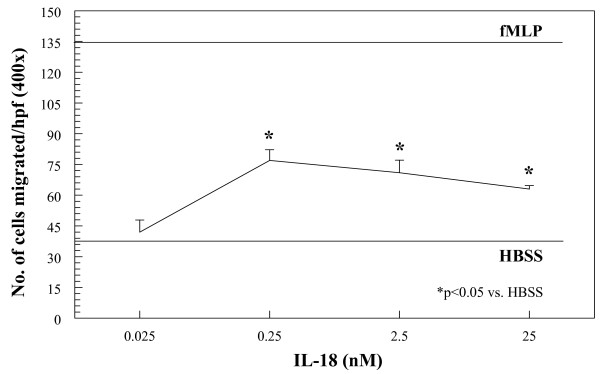
**Monocytes were isolated from the peripheral blood (PB) of normal (NL) volunteers and placed in a modified Boyden chemotaxis system opposite graded increases in concentration of rhuIL-18**. As shown, IL-18 stimulates chemotaxis for human monocytes in a dose-dependent manner, and is maximal between 0.25 n*M *and 25 n*M *(figure representative of three separate experiments).

Monocytes were isolated from the PB of NL volunteers and tested for migratory activity in a modified Boyden chemotaxis system. Figure 1 shows that monocytes readily migrate toward recombinant human IL-18 in a dose-dependent fashion, starting at 0.25 n*M *up to 25 n*M*. This indicates that IL-18 is chemotactic at concentrations similar to those found in RA SF [5].

### IL-18 signals via p38 and ERK½ in monocytes

**Figure 2:**
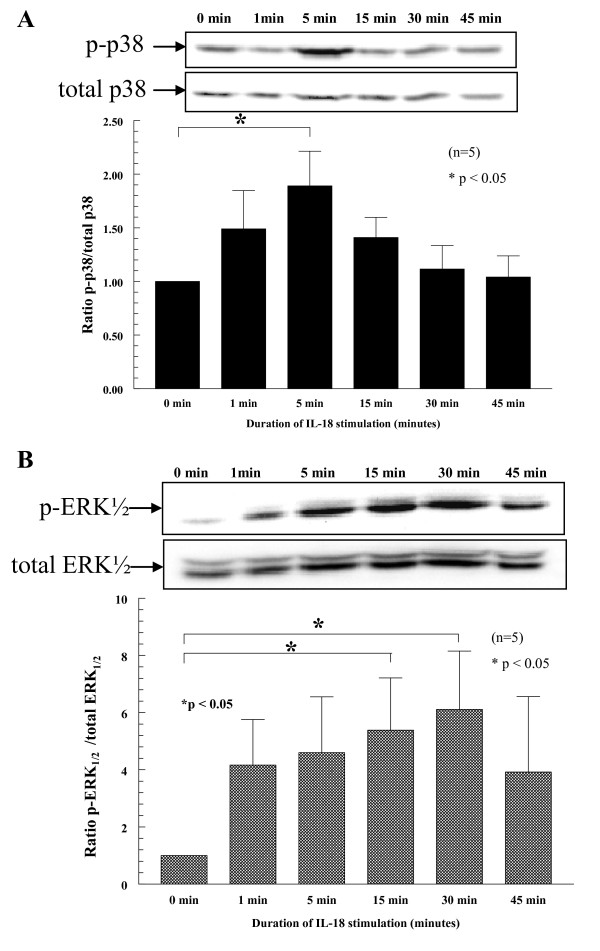
**IL-18 activates p-p38 and p-ERK½ in a time-dependent manner**. Monocytes (5 × 10^6 ^cells) were stimulated with 2.5 n*M *rhuIL-18. Cell lysates were made and probed for p-p38 and p-ERK½ with Western blot, showing marked increases in phosphorylation after 5 minutes for p-p38 and 15 to 30 minutes for p-ERK½. Representative blots show both p-p38 and p-ERK½ (upper panel for p-p38 and lower panel for p-ERK½). Graphs for p-p38 and p-ERK½ were normalized by respective total cellular expression for both signaling molecules relative to the untreated control blots (*n* = the number of blood donors, and graphs show combined data from five separate experiments). In total, five separate experiments were completed by using peripheral blood monocytes from four separate volunteers.

To define the kinetics of monocyte signaling pathways due to IL-18 stimulation, we used Western blots and examined four signaling pathways. Pathways tested were Jak2, JNK, p38, and ERK½. As shown in Figure 2, p-p38 was upregulated early at 5 minutes after IL-18 stimulation (upper panel). The effect was lost thereafter. p-ERK½ was upregulated by 15 minutes and showed maximal expression by 30 minutes (lower panel). Other signaling pathways, including Jak2 and JNK, were examined, but showed no significant expression resulting from IL-18 stimulation (data not shown). Graphs for p-p38 and p-ERK½ were normalized by respective total cellular expression for both signaling molecules relative to the untreated control blots.

From these findings, IL-18 appears to stimulate monocytes through the p38 and ERK½ pathway, suggesting that disruption of this pathway could mediate IL-18 stimulatory activity on monocyte function. Blots were normalized to total p38 and ERK½, respectively (representative blots shown). In total, five separate experiments were completed by using PB monocytes from four separate volunteers.

### Inhibition of p38 and ERK½ by ODN tranfection reduces monocyte chemotaxis to IL-18

**Figure 3:**
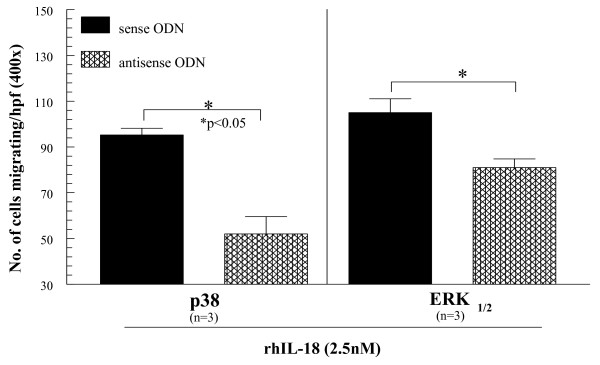
**Monocytes were suspended at 2.5 × 10^6 ^cells/ml and then transfected with sense or antisense ODNs in serum-free media for 4 hours**. Transfection efficiency for all genes was routinely > 80%, as determined by counting fluorescein isothiocyanate (FITC)-transfected cells with fluorescence microscopy and comparing with a DAPI label in the same cells (data not shown). Transfected cells were added to Boyden chemotaxis chambers to determine their migratory activity toward rhuIL-18 (2.5 n*M*). As shown, monocytes transfected with either antisense p38 or ERK½ showed significant reductions in chemotaxic activity toward rhuIL-18 compared with sense transfected cells (*n* = number of experimental repeats from independent PB monocyte donors).

We wanted to link signal-transduction pathways to monocyte function as a result of IL-18 stimulation. To do this, we inhibited both the p38 and ERK½ pathways with ODNs to each signaling molecule. Anti-sense ODN knockdown efficiency of intended targets was confirmed, as previously described [61]. We then tested the ability of rhuIL-18 (2.5 n*M*) to recruit PB monocytes as it did previously (Figure 1). As shown, transfection of monocytes with either antisense p38 or ERK½ significantly reduced the monocyte chemotactic activity of IL-18 compared with sense (nonspecific) ODN transfection (Figure 3). Jak2 and JNK were similarly inhibited but did not result in reductions of IL-18-stimulated monocyte chemotaxis (data not shown).

### IL-18 induces monocyte recruitment to synovium and LNs in the RA ST SCID mouse chimera

**Figure 4:**
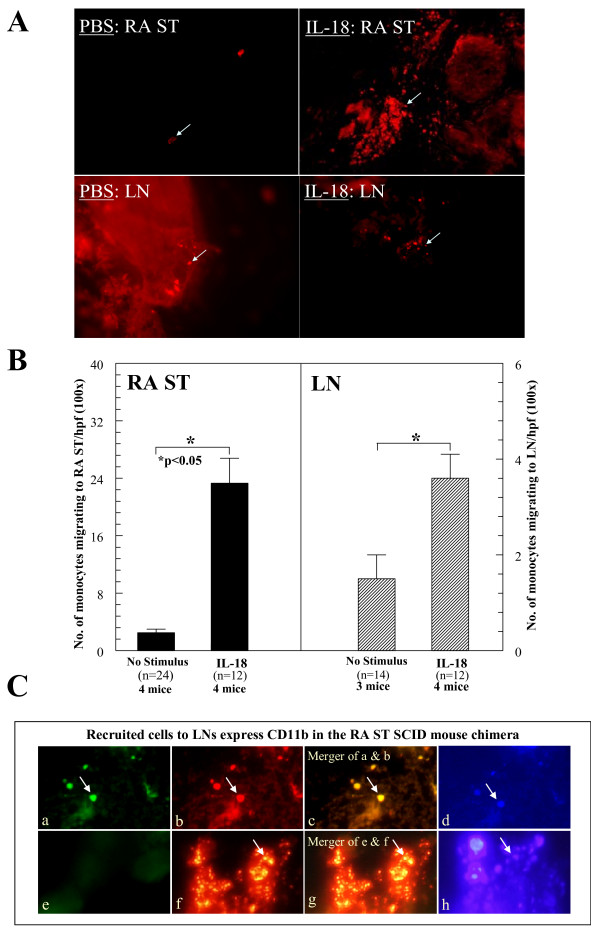
**Peripheral blood monocytes injection**. **(A)** PKH26 red fluorescent dye-tagged human peripheral blood (PB) monocytes (5 × 10^6^) were injected i.v. into SCID mice engrafted for 4 to 6 weeks with human rheumatoid arthritis synovial tissue (RA ST). Before administering cells, ST grafts were injected with rhuIL-18 (1,000 ng/graft) or sham injected (PBS stimulus). At 48 hours, grafts and inguinal lymph nodes (LNs) were harvested, and tissue sections were examined with immunofluorescence microscopy at 550 nm (100 ×). The top panel shows PKH26 dye-tagged monocytes migrating into PBS or rhuIL-18 injected RA ST. **(B) **The lower portion of the same panel shows an image of the local LNs containing recruited monocytes from the same mice. The number of dye-tagged cells migrating to engrafted RA ST or LN tissue in response to rhuIL-18 is graphed in the next panel. As shown, SCID mice receiving intragraft injections of rhuIL-18 showed significant recruitment of human monocytes to both engrafted RA ST and murine LNs. Monocyte migration was quantified by dividing the number of cells per hpf/tissue section at 100 × (*n* = number of tissue sections counted ± SEM). **(C) **LNs from rhuIL-18 simulated SCID chimeric mice were harvested and evaluated for human monocyte recruitment. LNs were stained for CD11b/Mac-1 with fluorescence histology. The primary antibody was a mouse anti-human mAb, followed by blocking with goat serum and the addition of a goat anti-mouse FITC-tagged secondary antibody. **(a) **Human monocytes expressing CD11b/Mac-1 migrate to murine LNs (fluorescent green cells, see arrow). **(b) **Fluorescent dye-tagged human cells in murine LNs. **(c) **Merger of **(a) **and **(b)**, showing that the migrating cells are expressing human CD11b/Mac-1 (fluorescent yellow staining; see arrow). **(d) **DAPI staining showing cell nuclei (fluorescent blue cells, see arrow). **(e) **Negative-control staining for CD11b/Mac-1 (nonspecific IgG was used as the primary mAb). **(f) **Murine LN showing recruited cells (red fluorescent staining, see arrow). **(g) **Merger of **(e) **and **(f) **showing a lack of nonspecific cellular staining. **(h) **DAPI staining showing cell nuclei (original magnification, 400 ×).

To test monocyte migration *in vivo*, we used an RA ST SCID mouse chimera model. After 4 to 6 weeks, animals engrafted with human RA ST showing no signs of rejection were used, as done previously [61]. To determine homing of NL human PB monocytes to RA ST *in vivo*, freshly isolated cells were fluorescently dye-tagged with PKH26, and 5 × 10^6 ^cells/100 μl/mouse were injected i.v. (tail vein) 48 hours before killing. Immediately after administration of dye-tagged cells, engrafted SCID mice received intragraft injections of rhIL-18 (1 μg/ml) or an equal volume of PBS. After 2 days, RA ST grafts and murine inguinal LNs were removed, and cryosections of tissues (10 μm) were examined by using a fluorescence microscope. The total number of mice used is indicated on the graph, with the "n" corresponding to the total number of sections analyzed from each treatment group. At least 12 sections/group, representing grafts taken from all the mice, were evaluated. Results from each section were average and divided by the number of hpfs (100 ×), to determine the number of migrating cells/hpf, as done previously [61]. Care was taken to represent each graft as equally as possible. Results are shown in Figure 4(a). IL-18, when administered intragraft, induced robust monocyte recruitment to both the RA ST grafts and local LNs (see arrows). In (b), graphs of both the RA ST and LN data clearly show that mice receiving IL-18 intragraft injections had significantly increased numbers of monocytes recruited to both implanted RA ST and local murine LN tissue in the SCID chimera system. In (c), to confirm that migrating cells to murine LNs were human monocytes, LNs from rhuIL-18-simulated SCID chimeric mice were harvested and evaluated for human monocyte recruitment. LNs were stained for CD11b/Mac-1 with fluorescence histology. The primary antibody was a mouse anti-human mAb, followed by blocking with goat serum and the addition of a goat anti-mouse FITC-tagged secondary antibody. (a) Human monocytes expressing CD11b/Mac-1 migrate to murine LNs (fluorescent green cells, see arrow). (b) Fluorescent dye-tagged human cells in murine LNs. (c) Merger of (a) and (b) showing that the migrating cells are expressing human CD11b/Mac-1 (fluorescent yellow staining, see arrow). (d) DAPI staining showing cell nuclei (fluorescent blue cells, see arrow). (e) Negative control staining for CD11b/Mac-1 (non-specific IgG was used as the primary mAb). (f) Murine LN showing recruited cells (red fluorescent staining, see arrow). (g) Merger of (e) and (f) showing a lack of nonspecific cellular staining. (h) DAPI staining showing cell nuclei (original magnification, 400×). Murine LN tissues were similarly stained for human CD4 and CD19 expression, but were negative for staining (data not shown).

### *IL-18 *gene-knockout mice have reduced ZIA-induced joint inflammation compared with Wt mice

**Figure 5:**
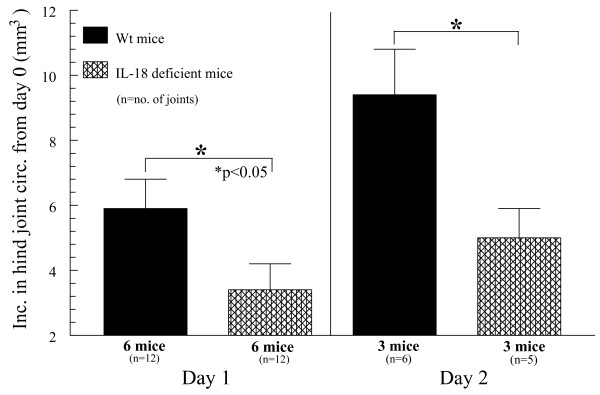
**Wt and *IL-18 *gene-knockout mice were administered zymosan to induce zymosan-induced arthritis (ZIA)**. Wt mice showed increases of hind joint (knee) circumference from 24 to 48 hours, with a pronounced reduction of swelling in comparative mice lacking *IL-18*. These data show that *IL-18 *is critical in acute inflammation of murine joints in as early as 24 hours after zymosan injection (*n* = number of joints analyzed).

The better to define the activity of *IL-18 *to induce inflammatory responses in acute models of arthritis, we administered to both Wt and *IL-18 *gene-knockout mice a single intraarticular (i.a.) injection of zymosan, inducing ZIA over a 48-hour period. Mice were divided into two separate groups and killed at either 24 or 48 hours. All mice were examined for joint swelling 24 hours later, and a smaller cohort containing the remainder of the mice was examined at 48 hours. *IL-18 *gene-knockout mice showed significant reductions of joint swelling as early as 24 hours, and this continued for up to 48 hours after ZIA induction (Figure 5). Notable increases in joint swelling were observed in both the Wt and *IL-18 *gene-knockout groups at 48 hours compared with 24 hours, with IL-18 deletion profoundly reducing joint swelling compared with that in Wt mice at both time points. These data suggest that *IL-18 *is produced early in the course of arthritic inflammation, indicating that it may be essential for stimulation of a proinflammatory cytokine cascade during acute inflammatory responses.

### Cytokine expression from sera and joint homogenates of ZIA mice

**Figure 6:**
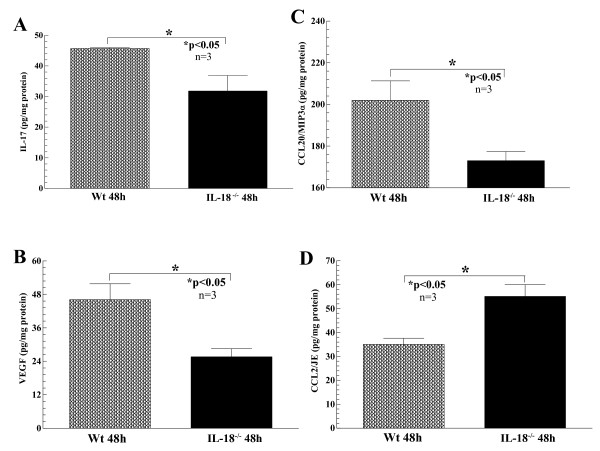
**Joint homogenates were prepared from both Wt and *IL-18 *gene-knockout mice injected with zymosan to induce zymosan-induced arthritis (ZIA)**. All tissue homogenates were initially measured for total protein content to normalize cytokine expression to total protein content for comparison between cytokines. Cytokines measured included IL-1β IL-6, IL-17, TNF-α MCP-1/CCL2, MIP-1α/CCL3, MIP-3α/CCL20, RANTES/CCL5, and VEGF. Although all cytokines measured were detectable in all the tissue homogenates, significant decreases of IL-17 **(a)**, VEGF **(b)**, and MIP-3α/CCL20 **(c) **were found in the *IL-18 *gene-knockout homogenates compared with Wt mice. Conversely, MCP-1/CCL2 **(d) **was significantly increased in the same homogenates from *IL-18 *gene-knockout compared with Wt mice (*n* = number of joints examined)

After killing, ZIA mouse serum and joints were harvested, and joint tissue was homogenized. Joint homogenates were measured for total protein content and assayed with ELISA for cytokines, including IL-1β IL-6, IL-17, TNF-α monocyte chemotactic protein-1 (MCP-1)/CCL2, macrophage inflammatory protein-1α MIP-1α/CCL3), MIP-3α/CCL20, regulated on activation normally T-cell expressed and secreted (RANTES)/CCL5, and vascular endothelial cell growth factor (VEGF). For comparisons, all cytokines measured were normalized to the total protein content of each homogenate. As shown in Figure 6, all mice showed detectable levels in joint homogenates of all cytokines tested; however, ZIA IL-18 gene-knockout mice showed significant reductions in IL-17 (a), VEGF (b), and MIP-3α/CCL20 (c) compared with ZIA Wt mice, indicating that expression of *IL-18 *can initiate proinflammatory cytokine release in joints during acute arthritis. Alternatively, homogenates from *IL-18 *gene-knockout mice increased MCP-1/CCL2 (JE) levels (d) due to zymosan injection compared with Wt mice, indicating that the expression of some monocyte recruitment factors may actually be inhibited because of the presence of IL-18. Sera from all groups of mice showed no significant differences in cytokine levels tested between the Wt and *IL-18 *gene-knockout mice induced for ZIA.

## Discussion

Our data show that IL-18 recruits monocytes *in vivo*, may be produced early in the acute phase of arthritis, and signals via p38 and ERK½ to recruit PB monocytes to STs. IL-18 is known to function in an autocrine or paracrine fashion, and increased expression of IL-18 in the synovium may play a critical role for development of synovial inflammation, synovial hyperplasia, and articular degradation to which angiogenesis may contribute [37]. Given the importance of angiogenesis in the pathophysiology of RA, we previously demonstrated a role for IL-18 as an angiogenic mediator [37]. Supportive of this function was the finding that IL-18 has been shown to stimulate production of angiogenic TNF-α [37,63].

We previously examined the signal-transduction mechanisms by which IL-18 induces vascular cell adhesion molecule-1 (VCAM-1) expression in RA synovial fibroblasts [31]. In that study, we outlined how IL-18 signals through the IL-18R complex composed of both α and β chains. Concerning the IL-18R complex, the IL-18Rα chain is the extracellular binding domain, whereas the IL-18Rβ is the signal-transducing chain. When bound to the IL-18R, IL-18 induces the formation of an IL-1R-associated kinase (IRAK)/TNF receptor-associated factor-6 (TRAF-6), a multipart structure that has stimulatory activity for nuclear factor κB (NF-κB) in Th_1 _cells [47] and in EL4/6.1 thymoma cells [31,64]. From our previous findings, we demonstrated that IL-18 induces VCAM-1 expression through Src kinase, PI3-kinase/Akt, and ERK½ signaling pathways [31], and outlined the participation of the IRAK/NF-κB pathway in RA synovial fibroblast VCAM-1 expression.

Dinarello and colleagues [65] showed that distinct differences exist in IL-1 and IL-18 signaling in transfected human epithelial cells, and that IL-1 signaling is primarily through the NF-κB pathway, whereas *IL-18 *signals via the MAPK p38 pathway. This finding may account for the absence of cyclooxygenase from *IL-18*-stimulated human epithelial cells and may explain the inability of IL-18 to induce fever, unlike IL-1 [65]. These findings also support our current signaling data showing that IL-18 induces p38 and ERK½ pathways in monocytes, confirmed by signaling inhibitory studies, Western blotting, and kinetic analysis showing that p38 is upregulated early in monocytes stimulated by IL-18, with subsequent upregulation of ERK½.

We also investigated a novel function of IL-18 to recruit monocytes *in vitro *and *in vivo*. Our *in vitro *data showed IL-18 chemotaxic activity for monocytes at levels of IL-18 similar to those found in RA SF [5]. We previously evaluated the role of IL-18 as an angiogenic mediator and showed that HMVECs respond to rhuIL-18 in a modified Boyden chemotaxis system [37]. For the current study, we purchased the rhuIL-18 from the same vendor with the exact specifics regarding sample purity. Our monocyte chemotaxis findings correlate well with other studies showing IL-18 to be chemotactic for human T cells and dendritic cells [66,67]. We also showed that at elevated levels beyond that found in the RA SF, IL-18 appears to be inhibitory for monocyte migration, similar to what we found in previous studies investigating MIP-3α and CXCL16 [35,61]. This is likely due to a regulatory feedback loop tempering cytokine function in acute and chronic inflammatory responses.

We then attempted to link the signaling data with *in vitro *monocyte migration findings by inhibiting monocyte p38 and/or ERK½ with ODNs, and then tested monocyte migratory activity toward IL-18 in a modified Boyden chemotaxis system. We show that disruption of IL-18-induced monocyte signaling using antisense ODNs confirmed our earlier observations of induced monocyte p38 and ERK½ activation by IL-18, resulting in significantly reduced monocyte chemotaxis. Although we did not demonstrate a direct effect of IL-18 by inhibition of downstream kinases, we did show that inhibition of kinases activated by IL-18 can alter monocyte migration toward IL-18 in a dose-dependent manner.

From these *in vitro *findings, further examination of the contribution of IL-18 in monocyte chemotaxis in an SCID mouse chimera system was warranted. To do this, SCID mice engrafted with RA ST received intragraft injections of rhuIL-18 with immediate administration of PB monocytes isolated, fluorescently dye tagged, and injected i.v. into chimeric mice, as done previously [61]. In this setting, IL-18 proved to be a robust monocyte chemotactic agent, directing migration of human monocytes not only to engrafted ST, but also to local (inguinal) murine LNs.

Data from the SCID mouse chimera provided circumstantial evidence that IL-18 may be an effective monocyte recruitment factor in chronic diseases and supported previous findings that IL-18 gene-knockout mice have reduced inflammation in relevant models of RA [1]. Rodent models of arthritis are indeed useful tools for studying the pathogenic process of RA. Although no model perfectly duplicates the condition of human RA, they are easily reproducible, well defined, and have proven useful for development of new therapies for arthritis, as exemplified by cytokine-blockade therapies. Furthermore, time-course studies consistently found that IL-1β, IL-6, TNF-α and other key pro-inflammatory cytokines and chemokines are functional in a variety of models, including CIA, adjuvant induced arthritis (AIA), SCW, and immune complex arthritis [68].

Notably, proinflammatory IL-18 activity has been extensively examined in CIA, an accepted animal model of RA, as it shares many immunologic and pathologic features of human RA [68]. This model is reproducible in genetically susceptible strains of mice with major histocompatibility haplotypes H-2^q ^or H-2^r ^by immunization with heterologous type II collagen in Complete Freund's Adjuvant. Susceptible strains are DBA/1, B10.Q, and B10.RIII [68]. Drawbacks of this model are that, in some studies, roughly a third of the mice do not develop arthritis, inherent inconsistencies in CIA progression, and that murine CIA can take a substantial time to develop, sometimes as much as 6 to 8 weeks. In addition, many gene-knockout strains are available only on the C57BL/6 background, a strain resistant to development of CIA. Despite the many hurdles, IL-18 has been shown to play a central role in CIA [1,50,69,70]. When injected into DBA-1 mice immunized with collagen in incomplete Freund's adjuvant, IL-18 increased the erosive and inflammatory component of the condition [1,5]. Using mice deficient in IL-18, CIA was less severe compared with Wt controls [1,50], and histologic evidence of decreased joint inflammation and destruction also was observed, outlining a direct pathologic role for IL-18.

We chose to use the ZIA model to examine the participation of IL-18 to induce a cytokine cascade by using IL-18 gene-knockout mice. Murine ZIA was first characterized by Keystone in 1977 [71]. This model is simple and straightforward, with arthritis induction initiated by a single i.a. injection of zymosan. Of note is that ZIA apparently lacks significant lymphocyte involvement and is therefore not well suited for experiments designed for examining T-cell or B-cell function in arthritis development. ZIA was chosen for this study primarily because of the timeliness of the inflammatory response and because IL-18, a monokine, is not known to be highly dependent on lymphocyte activation.

Zymosan is a polysaccharide from the cell wall of *Saccharomyces cerevisiae*. Zymosan is composed primarily of glucan and mannan residues [72,73]. *In vitro*, it has served as a model for the study of innate immune responses, such as macrophage and complement activation [74,75]. Zymosan is also recognized and phagocytosed principally by monocytes and macrophages and leads to cellular activation and monokine production [76], a nice feature when examining the participation of a monokine *in vivo*. The subsequent inflammatory response is thought to be mediated by activation of the alternative pathway of complement and the release of lysosomal hydrolases from activated macrophages [77]. Increasing evidence suggests that Toll-like receptors may also be involved [72]. The advantages of ZIA include its simplicity and the fact that the resultant inflammation it induces is not strain specific. ZIA also affords the opportunity to investigate cytokines involved in joint inflammation during an acute response that may offer insight into early proinflammatory cytokine release in the initial phases of the inflammatory response. This is often lost by using models such as CIA that normally take weeks to develop [78].

Using ZIA, we observed significantly reduced joint inflammation in IL-18 gene-knockout mice in as little as 24 hours after zymosan injection, and this trend continued for up to 48 hours.

We also found many proinflammatory cytokines similarly reduced in the joint homogenates of IL-18 gene-knockout mice, including IL-17, MIP-3α/CCL20, and VEGF. Although, as indicated earlier, ZIA is not known to be highly T-cell dependent, surprisingly, we found significantly reduced IL-17 and MIP-3 CCL20 in IL-18 gene-knockout ZIA joint homogenates, consistent with previous findings of CCR6, the MIP-3α/CCL20 receptor, located on T helper_17 _(Th_17_) lymphocytes [35,79] This interesting finding suggests that during certain acute joint-inflammatory models, T-cell subsets may become activated and express proinflammatory lymphokines. It is tempting to speculate that during an acute inflammatory response, Th_17 _cell subsets are activated and recruited to the joint, which may explain the increase in IL-17 in the joint homogenates of ZIA mice. This leads to the intriguing possibility that IL-18 may regulate Th_17 _responses by directly supporting MIP-3α/CCL20 and IL-17 expression in STs.

Also of note were the increased MCP-1/CCL2 levels in joint homogenates from ZIA IL-18 gene-knockout mice. This seemingly paradoxic finding can be explained by noting that IL-18 may induce expression of an unidentified MCP-1/CCL2 inhibiter, much like the association of TNF-α and IL-1-receptor antagonist protein (IL-1Ra). In the latter system, it has been demonstrated in both murine type-1 chronic pulmonary inflammatory models and from treatment of RA patients with a chimeric monoclonal antibody to TNF-α that TNF-α expression supports inhibition of IL-1β by upregulation of IL-1Ra, a natural antagonist of IL-1β [29,80]. We envision a similar scenario involving a regulatory loop for IL-18 and MCP-1/CCL2, as disruption of IL-18 significantly increased local expression of MCP-1/CCL2.

Detection of VEGF regulation was somewhat surprising, given the acute nature of ZIA. As ZIA is not normally thought of as a disease dependent on a high degree of vasculature, it should be noted that the inflammatory response observed in ZIA mice does indicate that proinflammatory cells are migrating freely from the peripheral bloodstream into joint tissues, presumably aided by additional vasculature mediated by VEGF. Furthermore, monocytes respond, produce, and migrate toward VEGF, providing further evidence that recruited monocytes may amplify the angiogenic process in acute inflammatory tissues expressing IL-18. It is tempting to speculate that the effects of VEGF on vasculature growth may exacerbate the IL-18-induced pathology seen in murine ZIA.

We found that IL-18 stimulates monocyte migration both *in vitro *and in an RA ST SCID mouse chimera system. We further show that this is mediated by activation of the p38 and ERK½ signaling pathway. We confirmed the latter finding by use of ODNs designed to disrupt this pathway and, in so doing, significantly reduced IL-18-mediated monocyte chemotaxis. We also showed that IL-18 gene-knockout mice have reduced ZIA, an acute model of RA, and that mice lacking IL-18 have significantly reduced joint homogenate levels of IL-17, MIP-3α/CCL20, and VEGF

Overall, this study indicates that IL-18 is effective very early in acute inflammatory models by inducing proinflammatory cytokine release and monocyte migration to STs, lending support to the notion that IL-18 plays a hierarchic role in the inflammatory cytokine cascade during arthritis development.

## Conclusions

We found that IL-18 stimulates monocyte migration both *in vitro *and in an RA ST SCID mouse chimera system. We further showed that this is mediated by activation of the p38 and ERK½ signaling pathway. IL-18 gene = knockout mice showed pronounced reductions in joint inflammation during ZIA compared with Wt mice, indicating that IL-18 may be produced in acute inflammatory responses and may serve a hierarchic position for initiating joint inflammatory responses.

## Abbreviations

IL-18: interleukin-18LN (lymph node); NL: normal; PB: peripheral blood; i.a.: intraarticular); RA ST: rheumatoid arthritis synovial tissue; rhu: recombinant human; SCID: severe combined immunodeficient; Th: T helper; Wt: wild type; ZIA: zymosan-induced arthritis.

## Competing interests

The authors declare that they have no competing interests.

## Authors' contributions

JHR, the first author, and CCP, the second author, designed and developed all aspects of the study, especially work with the RA ST SCID mouse chimera. JHR and HM performed the fluorescence histology on SCID LN tissue. MAA and CL assisted with arthritis induction and animal scoring of the ZIA model. CL and HM performed the Western blotting, and CL and JHR performed the chemotaxis studies. SS performed the ELISA assays on mouse ZIA joint homogenates. AEK, the senior author, was instrumental in the design and development of this project and generously offered her expertise.
